# Graphene Quantum Dots and Phthalocyanines Turn-OFF-ON Photoluminescence Nanosensor for ds-DNA

**DOI:** 10.3390/nano12111892

**Published:** 2022-05-31

**Authors:** Ana M. Santiago, Carla I. M. Santos, Leandro M. O. Lourenço, Inês F. A. Mariz, João P. C. Tomé, Ermelinda Maçôas

**Affiliations:** 1 Centro de Química Estrutural, Institute of Molecular Science and Departamento de Engenharia Química, Instituto Superior Técnico, Universidade de Lisboa, 1049-001 Lisboa, Portugal; ana.santiago@ist.utl.pt (A.M.S.); carla.santos@tecnico.ulisboa.pt (C.I.M.S.); inesfamariz@gmail.com (I.F.A.M.); jtome@tecnico.ulisboa.pt (J.P.C.T.); 2 LAQV-REQUIMTE and Department of Chemistry, University of Aveiro, 3810-193 Aveiro, Portugal; leandrolourenco@ua.pt

**Keywords:** sensor, graphene quantum dot, phthalocyanine, DNA, photoluminescence

## Abstract

Supramolecular hybrids of graphene quantum dots (GQDs) and phthalocyanine (Pc) dyes were studied as turn-OFF-ON photoluminescence nanosensors for detection of ds-DNA. Pcs with four (Pc4) and eight (Pc8) positive charges were selected to interact with negatively charged GQDs. The photoluminescence of the GQDs was quenched upon interaction with the Pcs, due to the formation of non-emissive complexes. In the presence of ds-DNA, the Pcs interacted preferentially with the negatively charged ds-DNA, lifting the quenching effect over the photoluminescence of the GQDs and restoring their emission intensity. The best performance as a sensor of ds-DNA was registered for the GQD-Pc8, with a limit of detection (LOD) in the picomolar range. The LOD for GQD-Pc8 was more than one order of magnitude lower and its sensitivity was about a factor of three higher than that of the analogue GQD-Pc4 nanosensor. The sensitivity and selectivity of this simple GQD-Pc8 nanosensor is comparable to those of the more sophisticated carbon-based nanosensors for DNA reported previously.

## 1. Introduction

The detection of biomolecules, such as nucleic acids and proteins, is of great interest for applications such as the early diagnosis of disease, industrial process control, and environmental monitoring. The singular characteristics of nanomaterials have placed them at the epicenter of several research projects with the aim of designing highly sensitive, selective, and low-cost biosensors [1,2]. Of particular interest are their large surface area and size tunable electrical, magnetic, and optical properties. To increase sensitivities and lower the detection limits, even to individual molecules, nanomaterials can immobilize a large quantity of biorecognition units at reduced volumes and, simultaneously, act as transduction elements that translate the interaction of the analyte with the recognition element into electrochemical, electro-chemiluminescent, magnetic, gravimetric, or optical signals. In this context, carbon-based nanomaterials such as graphene, graphene oxide, carbon nanotubes, nanodiamonds, and carbon dots have been intensively studied [2,3]. On the other hand, the combination of carbon-based nanomaterials with biomolecules such as DNA, sometimes mediated by metal nanoparticles, has also been extensively explored in biomedical applications, such as drug-delivery, gene therapy, and sensing [4,5].

Carbon dots are currently studied as optical biosensors, since they overcome several drawbacks of organic dyes and semiconductor quantum dots [3]. These materials are easy to synthesize and functionalize with organic molecules, and generally present interesting properties, such as high biocompatibility, good water solubility, low toxicity, low photobleaching, high fluorescence stability, and low environmental impact. Additionally, carbon dots have hydroxyl and carboxyl groups on their surface and edges, allowing covalent attachment, electrostatic interactions, and hydrogen bonding with other moieties [6]. The photoluminescence of carbon dots has been widely explored in bioimaging, diagnosis, bioanalytical assays, and biosensors [7]. Less common, but interesting, applications can be found in nanothermometry [8,9]. Reversible changes in their photoluminescence were used for the detection of ions, such as Hg^2+^ [10], Cu^+^ [11], CN^−^ [12], PO_4_^3−^ [13], or Cr^6+^ [14], and biomolecules, such as guanine [15], fructose [16], biothiols [17], ascorbic acid [14,18], DNA [19], and specific tumor markers (CA 15-3, CA 125, CA 19-9) [20,21,22]. Optical sensing using the photoluminescence of carbon dots follows a turn-OFF or turn-OFF-ON strategy, where either the presence of the analyte induces quenching of the photoluminescence of carbon dots, or their initially quenched photoluminescence is recovered in the presence of the analyte. For high sensitivity and selectivity, sophisticated carbon dot-based enzymatic sensors and carbon dot-based antigen–antibody constructs are needed [3].

Graphene quantum dots (GQDs) are a sub-type of carbon dots with a platelet-like structure composed by a limited number of sp^2^ carbon layers with delocalized π electrons, enabling strong π-π interactions with aromatic molecules. The photophysical properties of GQDs have been discussed in terms of quantum size effects, edge configuration and shape effects, chemical functionalities, heteroatom doping, and defects in the sp^2^ network [23,24,25,26]. More recently, the synergistic effects of the conjugation of GQDs with macrocycles, such as porphyrins [27,28], corroles [29], and Pcs [12,17,18,30], have been explored in the preparation of functional nanohybrids. The nanohybrid architecture afforded an improvement of the therapeutic performance as photosensitizers for photodynamic therapy [28,31], as well as the 3D-biomaging parameters, due to nonlinear excitation in the near-infrared and emission in the red part of the visible spectra [29]. They have also shown synergistic properties as catalysts for the oxygen reduction reaction [32], and good performance as turn-ON photoluminescent sensors [12,17,18].

**Figure 1:**
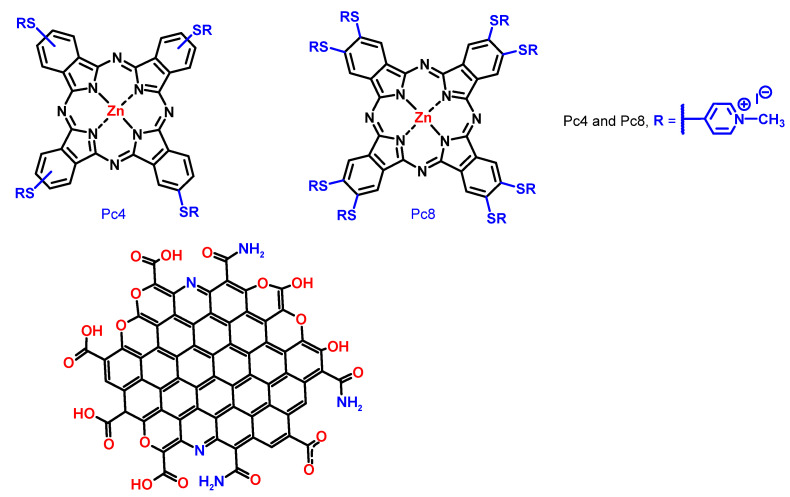
Structure of the Pc units and illustration of the structure of the GQDs in the nanocomposite.

Here, we prepared functional nanohybrids using GQDs and positively charged zinc-Pcs for sensing ds-DNA based on luminescence methods. The structures of the GQDs and the Pcs that composed the nanohybrids are shown in Figure 1. Notably, these Pcs are metallophthalocyanines that incorporate a Zinc atom at the center of a 16-membered ring formed by four isoindole units connected by four nitrogen atoms. They have a strong absorption in the red region of the UV-vis spectrum, with wavelengths above 670 nm; long triplet lifetimes (up to 1 ms); and high quantum yields of singlet oxygen generation (up to 0.7) [33]. Such photophysical properties have leveraged Pcs as second-generation photosentizers for antimicrobial and cancer photodynamic therapy [34,35,36,37]. Additionally, the Pc derivatives have been applied in electrochemical energy conversion and as storage devices [38], chemical sensors [39], and contrasting agents for magnetic resonance imaging (MRI) [40]. Moreover, Pcs can be easily conjugated with a variety of carbon-based materials, leading to an improvement of the photophysical and photochemical properties [41,42]. However, the interaction of charged Pcs and GQDs remains to a large extent unexplored; being addressed by less than a hand full of recent studies [43,44]. Loading of the Pcs onto the surface of GQDs is largely dependent on the charge of the Pc. Positively charged Pcs are adsorbed more efficiently onto the surface of negatively charged GQDs through electrostatic and π–π interactions [43]. The interaction between Pcs and GQDs can induce quenching of the photoluminescence of GQD, due to Förster resonance energy transfer (FRET) from the GQD to the Pc [30]. This interaction can increase the triplet quantum yield and singlet oxygen generation yield of the Pc [44,45].

In this study, GQD-Pc nanohybrids formed by electrostatic interactions are presented as a specific and sensitive approach for luminescence-based DNA sensing. Two supramolecular GQD-Pc nanohybrids were prepared, using Pcs with four (Pc4) and eight (Pc8) positive charges, as shown in Figure 1. The photoluminescence of the GQDs was quenched due to the formation of the non-emissive nanohybrids. In the presence of the ds-DNA, the Pcs interacted preferentially with the negatively charged ds-DNA, restoring the emission of the GQDs. The performance of the biosensor was dependent upon the charge of the Pc. Despite its simplicity, the LOD of the nanohybrids for ds-DNA (in the picomolar range) compared favorably with more sophisticated approaches based on carbon nanomaterials specially developed for luminescence sensing of DNA [46]. Electrochemical sensors based on carbon nanomaterials can reach lower levels of detection (down to the femtomolar range), but they are not suitable for in vivo detection [46,47].

## 2. Materials and Methods

### 2.1. Materials

Deoxyribonucleic acid sodium salt from salmon tests (Sigma-Aldrich, Darmstadt, Germany) and phosphate buffered saline tablets (PBS, Sigma-Aldrich, Darmstadt, Germany) were directly used as supplied. All aqueous solutions were prepared with ultra-pure water obtained from a Mili-Q Water system (Milipore Corp. Bedford, MA, USA). Interferents for the selectivity studies were L-cysteine (Cys, 97%, Sigma-Aldrich), DL-Homocystein (Hcy, 95%, Sigma-Aldrich), L-Glutathione reduced (GSH, 98%, Sigma-Aldrich), sodium azide (N_3_^−^, 99.5%, Sigma-Aldrich), sodium sulphate (SO_4_^2−^, ≥99%, Sigma-Aldrich), sodium thiosulfate (S_2_O_3_^2−^, ≥99.99%, Merck, Darmstadt, Germany), tetrabutylammonium iodine (I^−^, 98%, Sigma-Aldrich), tetrabutylammonium cyanide (CN^−^, 95%, Sigma-Aldrich), and tetrabutylammonium phosphate (PO_3_^−^, 99%, Sigma-Aldrich). 

### 2.2. Experimental Measurements

The UV-vis absorption spectra were recorded with a JASCO V-540 spectrophotometer (Jasco, Cremella, LC, Italy). The photoluminescence spectra were recorded in a Horiba Jobin Yvon Fluorolog 3–22 spectrofluorimeter (Horiba, Kyoto, Japan), equipped with a 450 W xenon lamp. The photoluminescence decays were followed by single photon timing using a Delta flex (Horiba, Kyoto, Japan) equipped with a 405 nm LED with a pulse width of 100 ps. The number of channels used to collect the emitted photons was 4096, with 53 ps per channel. The full width of the half maximum of the instrumental response function was measured as 200 ps, meaning that it was possible to measure lifetimes of the order of 20 ps. Deconvolution of the decay times was performed with TRFA Data Processor Advanced (v 1.5) developed by Scientific Software Technologies Center (Minsk, Belarus) using a multiexponential decay model. Typically, three exponentials were necessary to ensure a good quality of fit (χ^2^ ≤ 1.1 with the residuals randomly distributed around zero). All studies were performed based on a dispersion of 1 mg/mL of GQD in water. This dispersion was diluted to 0.05 mg/mL, corresponding to a concentration of ~2 µM of GQDs, for the titration experiments with the Pcs. For the Pcs, stock solutions of 50 µM in water were used. The molar concentration of double-stranded DNA (ds-DNA) was estimated using a molar mass of 1.3 × 10^6^ Da, corresponding to ~2000 base pairs (bp). Most results are discussed in terms of the base pair concentration. The Zeta potential was measured with Zetasiser nano ZS (Malvern Instruments, Malvern, UK). GQDs were dissolved in Milli-Q water and Pcs were dissolved in a mixture of water:THF (1:1).

### 2.3. Synthesis of GQDs and Pcs

The GQDs were prepared using a top-down approach, starting from graphite that was oxidized and chemically cut into nanographene oxide, as reported earlier [26]. Briefly, GQDs were obtained by a hydrothermal treatment of graphene oxide at 150 °C for 5 h in the presence of NH_4_OH and H_2_O_2_ [16]. The final product was filtered, and the supernatant was collected, dialyzed (1 kDA membrane) with deionized water for 7 days, and lyophilized. The concentration of GQDs was estimated using for the molar mass of the GQDs, with a value of 26 kDa corresponding to the molar mass of a cylinder of graphite (ρ = 2.25 g/cm^3^) with an average diameter of 4 nm and average height of 1.5 nm. The synthesis and characterization of the tetra- and octa-substituted thiopyridinium Pcs, Pc4 and Pc8, respectively, was previously reported on the literature [48].

### 2.4. Preparation of GQD-Pc4 and GQD-Pc8 Nanohybrids

The supramolecular GQD-Pc hybrids were obtained by electrostatic interaction between negatively charged GQD and positively charged Pcs. The hybrids were prepared at a concentration of 2 μM by mixing solutions of GQD (2 mM) and Pc4 or Pc8 (50 mM) in water in a molar proportion of 1:5 (GQD:Pc). The mixtures were freshly prepared one hour before measurements.

## 3. Results and Discussion

### 3.1. Characterization of GQDs

**Figure 2:**
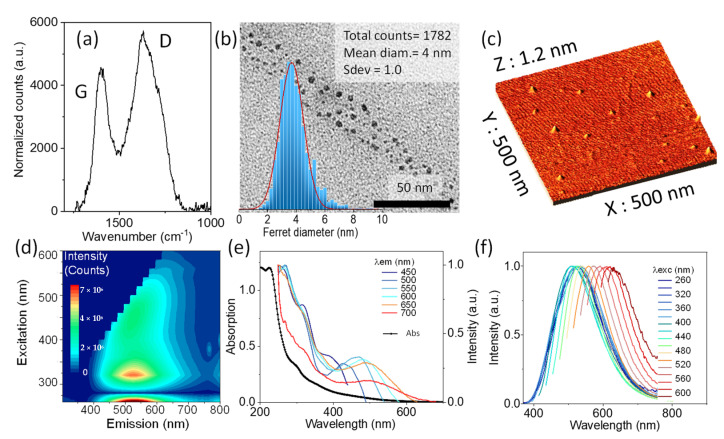
Characterization of the GQDs: (**a**) Raman spectra; (**b**) TEM image and histogram of Ferret diameter; (**c**) AFM image; (**d**) contour plot of excitation and emission; (**e**) absorption spectrum and excitation spectra collected at different emission wavelengths in the 450–700 nm range normalized at 250 nm; and (**f**) normalized emission spectra upon excitation in the range of 260–600 nm.

The diameter and height of the GQDs were measured by transmission electron microscopy (TEM) and atomic force microscopy (AFM), as reported earlier [26]. Representative TEM and AFM images are shown in Figure 2, together with the Raman spectrum. The average diameter and height were estimated to be 4 nm and 1.5 nm, respectively. The broad Raman spectrum of the GQD was characteristic of an amorphous carbon regime, with an average sp^2^ cluster size of 1.5 nm, derived from the intensity ratio of the D and G bands according with the Ferrari and Robertson relationship [49]. It is also important to mention that these GQDs showed a high percentage of oxygen (31%), in the form of hydroxyl and carboxyl groups that account for a net negative surface charge that favors interaction with the positive charges of Pcs. The zeta potential of the GQDs was measured to be −5 mV.

The optical characterization was performed in an aqueous dispersion of 0.05 mg/mL of GQDs corresponding to 2 µM of GQDs. The UV-vis absorption, photoexcitation, and photoluminescence emission spectra are shown in Figure 2. As many other carbon dots produced by top-down methods, these GQDs showed a broad absorption, extending from the ultraviolet to the red part of the visible spectrum (Figure 2e). The unstructured broad absorption is mainly connected with the structural heterogeneity of these GQDs, due to the presence of defects and functional groups at the surface and periphery, and also due to some distortion from planarity associated with the presence of such groups, conferring a certain conformational flexibility to the structure [26].

The emission spectrum was excitation wavelength dependent, with the maximum at 500 nm (Figure 2f). The structural heterogeneity of the GQDs seems to be the most trivial, but not the most popular, interpretation of this excitation dependent emission [24,26,50]. The emission yield was 10% at 440 nm [26]. The excitation spectrum was also emission wavelength dependent, and showed a maximum at 280 nm (Figure 2e). The mirror image relationship between the two suggests that the absorption and emission processes are localized in sp^2^ clusters, with no major charge or energy transfer occurring in the excited state. The contour plot of emission vs. excitation wavelength shows that there is also an excitation wavelength independent region below 300 nm (Figure 2d).

### 3.2. Characterization of Pc4 and Pc8

**Figure 3:**
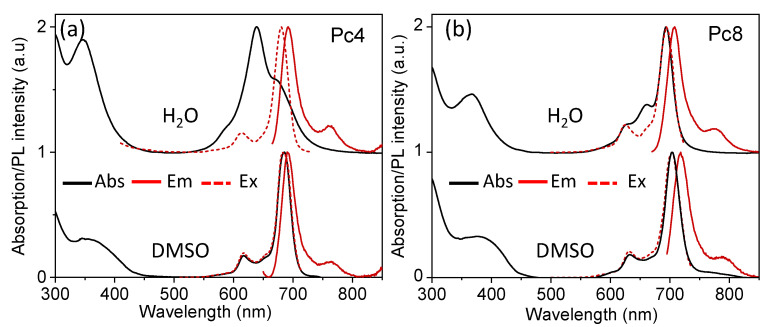
Optical properties of Pcs in water and DMSO: absorption, emission, and excitation spectra of (**a**) Pc4 and (**b**) Pc8.

The zeta potential of the Pcs was measured as +14 and +32 mV for Pc4 and Pc8, respectively; in good agreement with the relative amount of charge per molecule. The optical properties of the isolated Pcs were evaluated in different solvents, to determine the monomeric stability. The absorption, emission, and excitation spectra of Pc4 and Pc8 (5–10 µM) in DMSO and water solutions are shown in Figure 3. In DMSO, both Pcs showed the typical narrow NIR absorption of the Q-bands of monomeric zinc-Pcs and the slightly downshifted (lower energy) narrow emission from the same excited state. For the heavily charged Pc8, similar features were observed in aqueous solution, showing that the positive charges were effective in stabilizing the monomeric form. However, the Q-band of Pc4 broadened in water and the absorption maximum shifted to higher energies, revealing the predominance of non-emissive sandwich-like aggregates (H-type) formed by π-π stacking interactions. This observation suggests that the reduced charge density of Pc4 was not sufficient to stabilize the monomeric form in water. The charge is highly delocalized within the macrocycle and, thus, less effective in preventing aggregation in water. Nevertheless, the emission spectra of Pc4 in water showed only one narrow band, peaking at the same wavelength as the monomer band in DMSO (691 nm). The excitation spectrum collected at 760 nm confirmed that the emission originates from the monomeric Pc remaining in solution.

### 3.3. Titration of GQDs with Pcs

**Figure 4:**
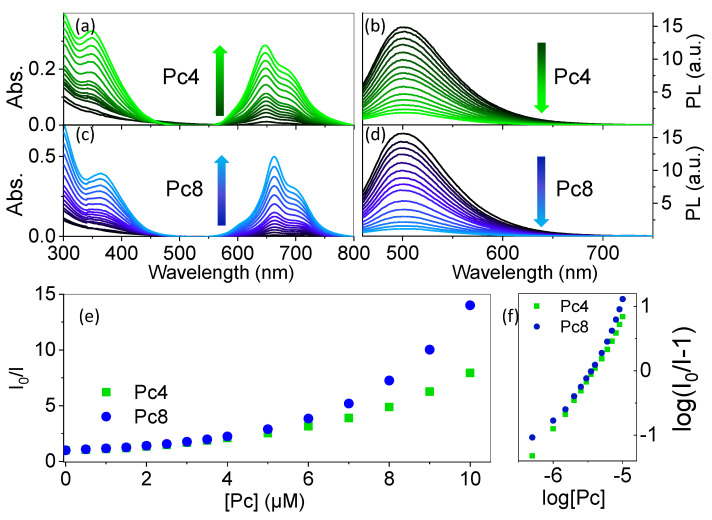
Absorption and emission of the aqueous dispersion of GQDs (2 μM, pH = 7.4) with increasing concentration of Pcs. Absorption spectra of the GQDs upon adition of (**a**) Pc4 and (**c**) Pc8; photoluminescence spectra of GQDs (λ_exc_ = 440 nm) upon addition of (**b**) Pc4 and (**d**) Pc8; (**e**) plot shows the ratio between the unquenched photoluminescence of GQD (I_0_) and photoluminescence of GQDs upon addition of the Pcs (I), and (**f**) is the log-log plot of the left- an right-hand side of Equation (4).

The formation of GQD-Pc hybrids was evaluated in aqueous solution upon titration of the GQDs dispersion (2 µM) with Pc4 or Pc8. The absorption and emission spectra were collected during the titration process (Figure 4). The emission of the GQDs at 500 nm decreased (λ_exc_ = 440 nm) concomitantly with the increase of the absorption of the Pcs. For both Pcs, the Q-bands appeared as broad structured bands, showing evident signs of interaction with the GQDs (Figure 4a,c). The emission of the Pcs was at best one order of magnitude lower than that of the GQDs, even at the highest concentrations of Pcs tested (Figure 4b,d), which is indicative of a strong quenching of the emission of the Pcs upon formation of the nanohybrid. The quenching of the emission of GQDs is presented in a Stern–Volmer-like plot (I_0_/I vs. quencher concentration) in Figure 4e, where I is the photoluminescence intensity. The upward curvature in the Stern–Volmer plot suggests that each GQD can interact with more than one Pc in an equilibrium described by the following equation:

(1) $$\mathrm{GQD}\;\left(\mathrm{aq}\right)+\mathrm{nPc}\;\left(\mathrm{aq}\right)⇋\mathrm{GQD}-{\mathrm{Pc}}_{n}\left(\mathrm{aq}\right)$$

In such case, considering that:

(2) $$K_{S}=\frac{{\left[\mathrm{GQD}-\mathrm{Pc}\right]}_{e}}{{\left[\mathrm{GQD}\right]}_{e}{\left[\mathrm{Pc}\right]}_{e}^{n}{}_{}}$$

(3) $${\left[\mathrm{GQD}-{\mathrm{Pc}}_{n}\right]}_{e}={\left[\mathrm{GQD}\right]}_{0}-{\left[\mathrm{GQD}\right]}_{e}$$

where $K_{S}$ is the static quenching constant and the subscripts *e* and *0* are relative to equilibrium and initial concentration, respectively, we would expect a linear correlation for the log-log plot of $\frac{I_{0}}{I}-1$, as a function of the concentration of Pc:

(4) $$\frac{I_{0}}{I}-1=K_{S}{\left[\mathrm{Pc}\right]}^{n}$$

where the slope would give the number of Pcs per GQD in the complex. However, as shown in Figure 4f, no such linear correlation was verified. The failure to describe the quenching mechanism using Equation (4), suggests that the mechanism of interaction of the GQDs with the Pcs is more elaborate, possibly involving the coexistence of complexes with different numbers of Pc per GQD in solution and also a contribution from dynamic quenching [43].

### 3.4. Sensing of DNA

**Figure 5:**
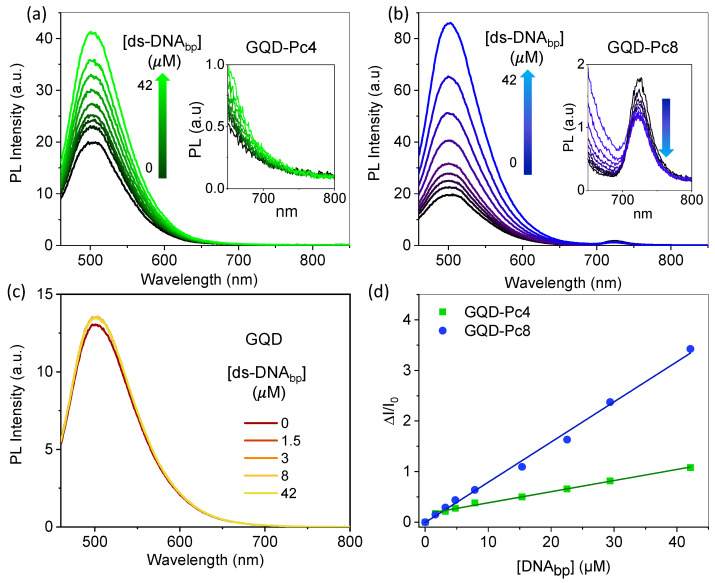
Photoluminescence response of the nanosensors (GQD-Pc4 and GQD-Pc8) and GQDs at a concentration of 2 μM in the presence of ds-DNA. The photoluminescence spectra of: (**a**) GQD-Pc4, (**b**) GQD-Pc8, and (**c**) GQD, upon addition of ds-DNA (in PBS, pH 7.4) up to a concentration of 42 μM; and (**d**) calibration curve obtained for the nanohybrid data described as ΔI/I_0_ = 0.164 + 0.022[ds*-*DNA_bp_ (μM)] for Pc4 and ΔI/I_0_ = −0.004 + 0.079[ds*-*DNA_bp_ (μM)] for Pc8, both with R^2^ = 0.995.

The emission of supramolecular GQD-Pc hybrids has been explored in the sensing of cyanide ions [12], biothiols [17], and ascorbic acid [18]. The rationale behind such approach is that the Pcs acting as a quencher of the emission of the GQDs (turn-OFF) in the nanosensor should preferentially interact with the analyte and promote the recovery of the emission (turn-ON) of the GQDs. Having such a strategy in mind, GQD-Pc nanohybrids have been explored as turn-OFF-ON sensors for ds-DNA. Titration experiments were performed to evaluate the sensitivity of the photoluminescence of GQD-Pc4 and GQD-Pc8 nanohybrids to the presence of ds-DNA. Figure 5 shows the systematic recovery of the photoluminescence of the nanohybrids with increasing concentration of ds-DNA. The sensitivity and limits of detection of the nanohybrids were evaluated from their fractional photoluminescence enhancement, using Equation (5):

(5) $$\frac{I-I_{0}}{I_{0}}=k\left[\mathrm{ds}-{\mathrm{DNA}}_{\mathrm{bp}}\right]+\mathrm{const}\;$$

A good linear correlation exists between the recovery of the photoluminescence of the GQDs and the concentration of DNA up to a concentration of 42 μM bp, corresponding to a ds-DNA molar concentration of ~20 nM (higher concentrations were not tested). The LOD was evaluated based on the regression line, considering only the lower concentrations in the plot (<7 μM bp) using the standard deviation of the y-intercepts (S_y_) and the slope (S) as LOD = 3S_y_/S. The obtained LOD for Pc8 was 66 nM bp, corresponding to a ds-DNA molar concentration of ~30 pM. This value compares rather favorably with the values of 25–75 pM recently reported for the best performing carbon-based nanomaterials developed for DNA biosensing, based on luminescent methods [46]. The obtained LOD for Pc8 was more than one order of magnitude lower (17 times) than the LOD for Pc4 (1.1 μM bp). The slope of the fractional photoluminescence enhancement plot was larger for Pc8 (7.9 × 10^4^ M^−1^) than for Pc4 (2.2 × 10^4^ M^−1^), revealing that a higher number of charges in the Pc favors the sensitivity towards ds-DNA.

### 3.5. Mechanistic Insight

**Figure 6:**
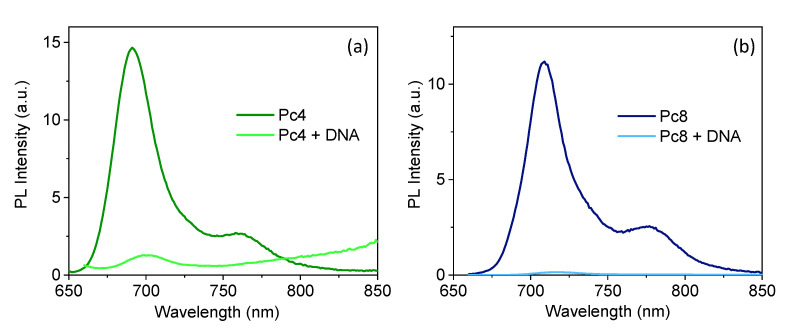
Quenching effect of ds-DNA on the photoluminescence of the Pcs (10 μM in water), showing a decrease of the photoluminescence intensity for (**a**) Pc4 and (**b**) Pc8 in the presence of ds-DNA (42 μM of base pairs at pH 7.4 in water).

Control experiments were performed to elucidate the nature of the interaction between ds-DNA and isolated Pcs or isolated GQDs. The effect of ds-DNA on the emission of isolated GQDs is shown in Figure 5c, while Figure 6 shows the effect of ds-DNA on the emission of isolated Pcs. No significant changes in the emission of GQDs were observed upon titration of GQDs with DNA. Conversely, the interaction of DNA promoted a significant quenching of the emission of the Pcs (Figure 6a,b). These two observations are evidence that ds-DNA interacts strongly with the Pc, due to the electrostatic attraction of their opposing charges, while no significant interaction exists between the ds-DNA and the GQDs, because they are both negatively charged. Further evidence of the preferential interaction of the Pcs with ds-DNA is provided by the fact that addition of ds-DNA to the GQD-Pc nanohybrid dispersion did not lead to a significant recovery of the emission of monomeric Pcs (inserts in Figure 5a,b). We can conclude that the turn-OFF-ON sensing mechanism of ds-DNA by the GQD-Pc was based on the recovery of the emission of the GQD in the presence of ds-DNA, due to disruption of the GQD-Pc non-emissive hybrid promoted by a higher affinity of the Pc with ds-DNA.

**Figure 7:**
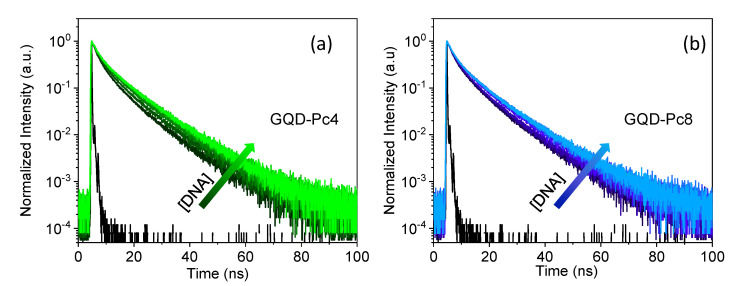
Effect of ds-DNA on the photoluminescence decay of the nanosensor for (**a**) GQD-Pc4 and (**b**) GQD-Pc8, upon addition of DNA up to a concentration of 200 μM of base pairs (the decays at 20, 40, 80, 120, 160, and 200 μM of DNA bp are shown).

A further insight into the mechanism of recovery of the photoluminescence intensity of GQD in the presence of ds-DNA is provided by the emission lifetimes. As shown in Figure 7, in both hybrids, the average emission lifetime of the GQDs increased systematically, from 3.8 up to 5 ns, with the concentration of DNA base pairs. This shows that the interaction of ds-DNA with the Pc not only allows recovery of emission, by decreasing the concentration of the GQD-Pc non-emissive hybrids, but also reduces the dynamic quenching contribution.

### 3.6. Selectivity Studies

**Figure 8:**
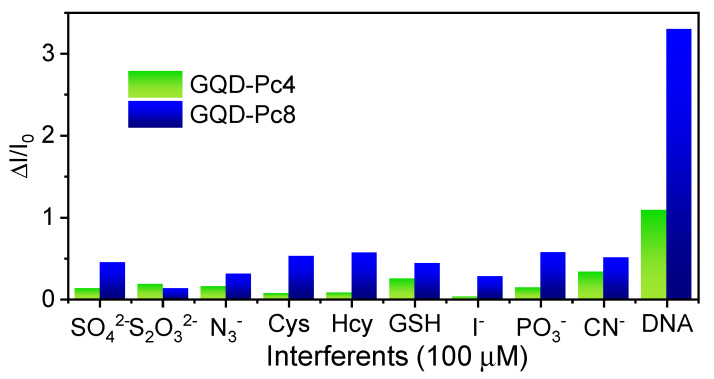
Selectivity of the nanosensors (GQDs-Pc4 and GQDs-Pc8 with 1:5 ratio of GQD:Pc and [GQD] = 2 μM) towards DNA, showing the photoluminescence enhancement in the presence of an excess (100 µM) of the various relevant analytes.

The selectivity of the nanosensors towards ds-DNA was tested against biothiols with net negative charge at pH = 7, such as cysteine (Cys), DL-homocysteine (Hcy), and glutathione (GSH), as well as some anions (SO_4_^2−^, S_2_O_3_^2−^, N_3_^−^, I^−^, PO_3_^−^ and CN^−^) as potential interferents. For both sensors, Figure 8 shows that the photoluminescence response in the presence of any such potential interferents was significantly lower than the one observed in the presence of ds-DNA. While in the presence of ds-DNA there was an obvious emission enhancement, none of the other interferents led to a significant turn-ON effect.

## 4. Conclusions

A specific and sensitive approach for luminescent DNA probes is presented based on the formation of GQD-Pc nanohybrids by electrostatic interactions. Despite its simplicity, the limit of detection for ds-DNA of ~30 pM compares favorably with the reported carbon-based nanomaterials specially developed for the luminescence sensing of DNA. The obtained limit of detection for Pc8 was more than one order of magnitude lower than the limit of detection for Pc4. The slope of the fractional photoluminescence enhancement plot was nearly four times larger for Pc8 than for Pc4, revealing that a higher number of charges in the Pc favors the sensitivity towards ds-DNA. Both nanosensors revealed a good selectivity when tested against biothiols with net negative charge and inorganic anions as potential interferents. The simplicity of the discussed approach, and its cost-effectiveness, can provide the necessary leverage for further performance improvements.

## Data Availability

The data presented in this study are available upon reasonable request from the corresponding author.
